# Dual anti-HIV mechanism of clofarabine

**DOI:** 10.1186/s12977-016-0254-0

**Published:** 2016-03-24

**Authors:** Michele B. Daly, Megan E. Roth, Laurent Bonnac, José O. Maldonado, Jiashu Xie, Christine L. Clouser, Steven E. Patterson, Baek Kim, Louis M. Mansky

**Affiliations:** Center for Drug Discovery, Department of Pediatrics, Emory Center for AIDS Research, Emory University, Children’s Healthcare of Atlanta, 1760 Haygood Dr., Atlanta, GA 30322 USA; Institute for Molecular Virology, University of Minnesota, 18-242 Moos Tower, 515 Delaware St SE, Minneapolis, MN 55455 USA; Department of Diagnostic and Biological Sciences, School of Dentistry, University of Minnesota, Minneapolis, MN 55455 USA; Department of Microbiology and Immunology, University of Minnesota, Minneapolis, MN 55455 USA; Center for Drug Design, Academic Health Center, University of Minnesota, Minneapolis, MN 55455 USA

**Keywords:** Human immunodeficiency virus (HIV), Reverse transcription, Ribonucleotide reductase, Clofarabine, Nucleoside/nucleotide analogue

## Abstract

**Background:**

HIV-1 replication kinetics inherently depends on the availability of cellular dNTPs for viral DNA synthesis. In activated CD4^+^ T cells and other rapidly dividing cells, the concentrations of dNTPs are high and HIV-1 reverse transcription occurs in an efficient manner. In contrast, nondividing cells such as macrophages have lower dNTP pools, which restricts efficient reverse transcription. Clofarabine is an FDA approved ribonucleotide reductase inhibitor, which has shown potent antiretroviral activity in transformed cell lines. Here, we explore the potency, toxicity and mechanism of action of clofarabine in the human primary HIV-1 target cells: activated CD4^+^ T cells and macrophages.

**Results:**

Clofarabine is a potent HIV-1 inhibitor in both activated CD4^+^ T cells and macrophages. Due to its minimal toxicity in macrophages, clofarabine displays a selectivity index over 300 in this nondividing cell type. The anti-HIV-1 activity of clofarabine correlated with a significant decrease in both cellular dNTP levels and viral DNA synthesis. Additionally, we observed that clofarabine triphosphate was directly incorporated into DNA by HIV-1 reverse transcriptase and blocked processive DNA synthesis, particularly at the low dNTP levels found in macrophages.

**Conclusions:**

Taken together, these data provide strong mechanistic evidence that clofarabine is a dual action inhibitor of HIV-1 replication that both limits dNTP substrates for viral DNA synthesis and directly inhibits the DNA polymerase activity of HIV-1 reverse transcriptase.

**Electronic supplementary material:**

The online version of this article (doi:10.1186/s12977-016-0254-0) contains supplementary material, which is available to authorized users.

## Background

Deoxyribonucleoside triphosphates (dNTPs) are essential for the genomic DNA replication of all organisms. In mammalian cells, two pathways supply the cell with dNTPs: the de novo synthesis pathway and the salvage pathway. Ribonucleotide reductase (RNR) is the rate-limiting enzyme in de novo dNTP synthesis and acts by reducing ribonucleotides to deoxyribonucleotides. Nucleotide levels and cell cycle status tightly regulate the expression and activity of RNR [1, 2]. Prior to S phase, RNR activity greatly increases ensuring a sufficient supply of dNTPs for DNA replication. However, cells that are in nondividing or resting states display restricted RNR activity, which results in a low dNTP environment [3, 4]. In contrast, transformed/cancer cells, which are rapidly dividing with uncontrolled cell cycles, have significantly higher dNTP levels compared to normal dividing cells, and elevated dNTP levels are considered a biochemical marker for cancer cells [4, 5].

Various intracellular pathogens that synthesize DNA, including human immunodeficiency virus type 1 (HIV-1), use cellular dNTPs for their genome replication. Two primary target cells of HIV-1 are activated CD4^+^ T cells and macrophages. Activated CD4^+^ T cells are dividing cells that contain abundant dNTPs (1–5 μM), synthesized by the highly expressed RNR, to support the replication of their genome [6–8]. On the other hand, macrophages are terminally differentiated and nondividing. Due in part to their extremely low RNR expression they have substantially lower dNTPs (20–50 nM) than dividing cells [7–9]. HIV-1 replication kinetics is slower in macrophages compared to activated CD4^+^ T cells [8, 10]. We previously reported that the low dNTP pools found in macrophages kinetically delays HIV-1 reverse transcription, suggesting that limited dNTPs serve as a restriction mechanism against HIV-1 in nondividing cells [8].

Recently, SAM domain and HD domain containing protein 1 (SAMHD1) was identified as a potent myeloid-specific host restriction factor of HIV-1 that depletes cellular dNTPs [11], which subsequently suppresses HIV-1 replication in nondividing cell types such as macrophages [12–14]. Interestingly, unlike HIV-1, HIV-2 and many SIVs encode a viral accessory protein, viral protein X (Vpx), that counteracts SAMHD1 and promotes retroviral replication in macrophages by elevating cellular dNTPs [13, 15, 16]. In addition to SAMHD1, other dNTP pool modulators have been shown to effect HIV reverse transcription. Most notably, cyclin-dependent kinase p21 inhibits HIV replication by repressing the expression of an alternative RNR subunit called RNR2 [17]. Overall, these data indicate that sufficient dNTP levels are necessary for viral reverse transcription and depleting dNTPs restricts viral replication.

**Fig. 1:**
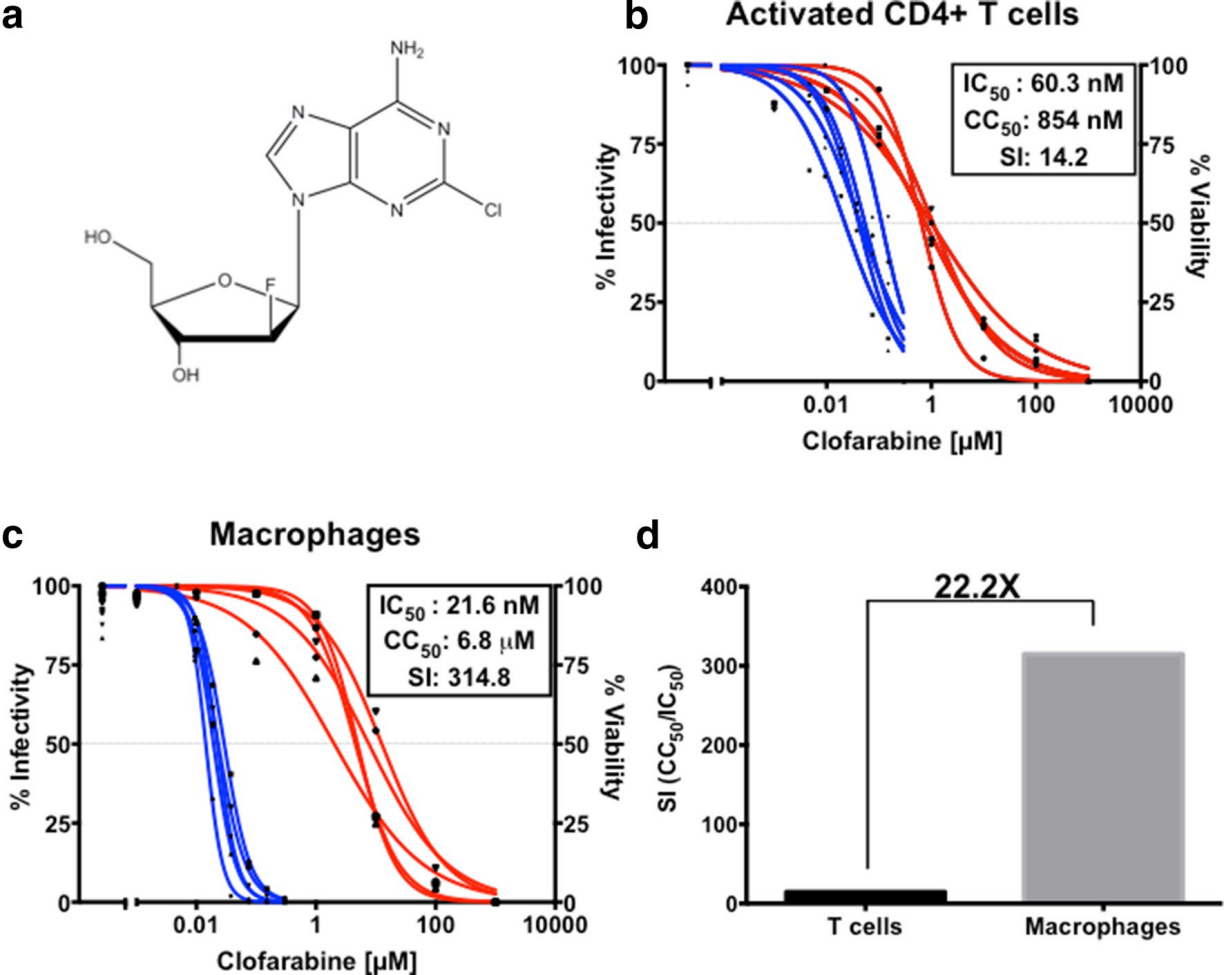
Anti-HIV-1 activity of clofarabine in primary human activated CD4^+^ T cells and monocyte derived macrophages. **a** The structure of clofarabine. **b** Clofarabine inhibition (*blue lines*) and cytotoxicity (*red lines*) on activated CD4^+^ T cells of 5 healthy donors. Cells were treated with increasing concentrations of clofarabine for 8 h, washed with PBS, and then infected with pseudotyped HIV-1, (inhibition) or cultured with media (cytotoxicity). Analysis was conducted at 72 h post-infection via flow cytometry (inhibition) or XTT assay (cytotoxicity). The IC_50_ is 60.3 nM with a 95 % confidence interval (95 % CI) of 24.1–96.5 nM; the CC_50_ is 854.2 nM with a 95 % CI of 712.6–995.8 nM. **c** The clofarabine inhibition (*blue lines*) and cytotoxicity curves (*red lines*) for monocyte-derived macrophages of 5 healthy donors. Macrophages were treated as described for T cells except analysis was at 5 days post infection. IC_50_ = 21.6 nM (95 % CI 17.4–25.8 nM): CC_50_ = 6.8 μM (95 % CI 3.2–9.4 μM). **d** Selectivity Index (SI) difference between activated CD4^+^ T cells and macrophages. SI values were determined by dividing the average CC_50_ of five donors by the average IC_50_ of five donors

Clofarabine is an FDA approved RNR inhibitor (RNRI) used in the treatment of acute lymphoblastic leukemia. As a purine nucleoside analog (Fig. 1a), clofarabine is transported into cells by nucleoside transporters and is phosphorylated by host enzymes to the active forms of the drug, clofarabine di- and triphosphate [18]. Both clofarabine di- and triphosphate inhibit RNR by binding the catalytic and the allosteric regulatory sites inducing hexamerization of the large subunit of RNR and subsequently preventing formation of the active enzyme. Due to the necessity of RNR in de novo dNTP synthesis, this inhibition causes a reduction in endogenous dNTPs, which in turn can inhibit DNA synthesis due to limited substrate availability [19, 20]. In addition to RNR inhibition, clofarabine triphosphate (clofarabine-TP) can also be incorporated as an adenosine analog by DNA polymerase-α and -ε and can induce chain termination [21, 22]. Other DNA polymerases can also incorporate clofarabine albeit at a much-reduced rate that is not of biological significance. The mechanism for chain termination of clofarabine is not entirely clear; as clofarabine has a 3′ OH, it is not an obligate chain terminator like azidothymidine (AZT). It has been observed that incorporation of clofarabine-TP reduces the rate at which the next nucleotide will be incorporated and incorporation of two consecutive clofarabine-TPs makes it extremely unlikely that the DNA chain will be further elongated. One current hypothesis for this inhibition of extension is that the 2′ fluorine atom may affect the reactivity of the 3′ OH and/or the quaternary structure of the DNA such that the polymerase fails to efficiently make the next bond [22].

We have previously demonstrated that clofarabine has anti-HIV activity in cell culture using a transformed cells line [23], however these studies did not examine the mechanism of action, or the anti-HIV activity and toxicity in the primary target cells of HIV: activated CD4^+^ T cells and macrophages. Our biochemical and cell culture data indicate that clofarabine effectively blocks HIV-1 replication by two distinct mechanisms: (1) reduction of cellular dNTPs and (2) direct incorporation by and inhibition of HIV-1 reverse transcriptase, with very limited toxicity in macrophages.

## Results and discussion

### Anti-HIV-1 activity and cytotoxicity of clofarabine in human primary target cells

Clofarabine, a purine nucleoside analog (Fig. 1a) and RNRI, is an FDA approved anticancer compound that we have recently shown to have antiretroviral potency in a transformed cell line [23]. Here, we examined the anti-HIV-1 activity of clofarabine in the primary human cells that are targets of HIV-1: activated CD4^+^ T cells and monocyte derived macrophages (MDMs). Cells were isolated from five healthy donors, pretreated with varying concentrations of clofarabine for 8 h, and then infected with HIV-1 pseudotyped with vesicular stomatitis virus G protein (VSV-G). The construct used expresses the full-length HIV-1 genome, with a frameshift in *env* and two fluorescent protein genes, *mCherry* and *enhanced GFP* (*EGFP*), replacing a portion of *rev* and *nef* [24]. Cells were analyzed with flow cytometry at 5 days (MDMs) or 3 days (T cells) after the addition of virus, and infected cells were determined by EGFP expression. Macrophages, as expected, showed a more restricted HIV-1 infection than the CD4^+^ T cells; however, however similar infectivity was achieved by using five times the amount of virus in MDMs (Additional file 1: Figure S1A). As shown in Figs. 1b and c (blue lines), clofarabine caused a concentration-dependent decrease in HIV-1 infection in both cells types, with half maximal inhibitory concentration (IC_50_) values of 21.6 nM [95 % confidence interval (95 % CI) 17.4–25.8 nM] in macrophages and 60.3 nM (95 % CI 24.1–96.5 nM) in activated CD4^+^ T cells. This three-fold increase in potency in macrophages compared to T cells is surprisingly minor—in the low dNTP environment of macrophages, we expected that the ratio of clofarabine-DP and -TP to dADP and dATP, respectively, would be much higher than that found in T cells, and therefore considerably more potent. However, this analysis is complicated by the fact clofarabine-TP has recently been identified as a substrate for SAMHD1, which is highly expressed in macrophages but not T cells [25].

We also determined the cytotoxicity of clofarabine in activated CD4^+^ T cells and macrophages (red lines in Fig. 1b, c) using the XTT assay, and found that macrophages are far more resistant to clofarabine-induced toxicity than activated CD4^+^ T cells, with CC_50_ values of 6.8 μM (95 % CI 3.2–9.4 μM) and 854 nM (95 % CI 713–996 nM), respectively. Additional toxicity assays, including analysis of membrane integrity and cell size, were performed and supported this result (Additional file 1: Figure S1B–E).

This eight-fold difference in cytotoxicity indicates that macrophages are significantly more resistant to the toxic effects of clofarabine. The difference in clofarabine toxicity in macrophages and T cells may be due to multiple factors. One possibility is that T cells are actively dividing which provides an opportunity for clofarabine-TP to be incorporated into their genome [26]. In cancer cells this genomic incorporation of clofarabine-TP has been show to be toxic. Additionally, nucleotide starvation due to RNR inhibition and DNA damage response can induce cell cycle arrest and potentially lead to apoptosis [27–29]. These factors would not necessarily affect macrophages because they are nondividing state and therefore not replicating their genome and macrophage nucleotide levels are already extremely low compared to dividing cells. Another possible explanation is that clofarabine-TP, along with other dATP analogs, is known to induce mitochondrial toxicity by altering the mitochondrial transmembrane potential [30]. SAMHD1, which is highly expressed in macrophage but not T cells, may be degrading clofarabine-TP and therefore limiting the effect of mitochondrial toxicity in MDMs.

Despite the fact that clofarabine-TP can be degraded by SAMHD1, clofarabine remains very potent in macrophages (IC_50_ = 20.3 nM) and has limited cytotoxicity in this cell type. The selectivity index (SI, CC_50_/IC_50_) for clofarabine in macrophages is 314.8, 22-fold greater than the SI in activated CD4^+^ T cells (Fig. 1d), suggesting that clofarabine is a highly selective inhibitor of HIV-1 specifically in macrophages.

### Effect of clofarabine on cellular dNTP levels and HIV-1 DNA synthesis

We previously reported that the dNTP concentration in activated CD4^+^ T cells (1–5 μM) is above the Km value of HIV-1 RT (100–200 nM) [8, 31]. On the other hand, macrophages have low dNTPs (50 nM) with concentrations that are below the Km value of HIV-1 RT, suggesting that the low dNTP levels kinetically delay HIV-1 reverse transcription in macrophages [8]. Clofarabine is a known RNRI that can deplete endogenous dNTPs in transformed cell lines [21, 22]. Here, we wanted to investigate the extent to which clofarabine depleted endogenous dNTPs in activated CD4^+^ T cells and macrophages. Specifically, we were interested in whether clofarabine would be effective in the extremely low dNTP environment in macrophages where RNR is not robustly expressed.

**Fig. 2:**
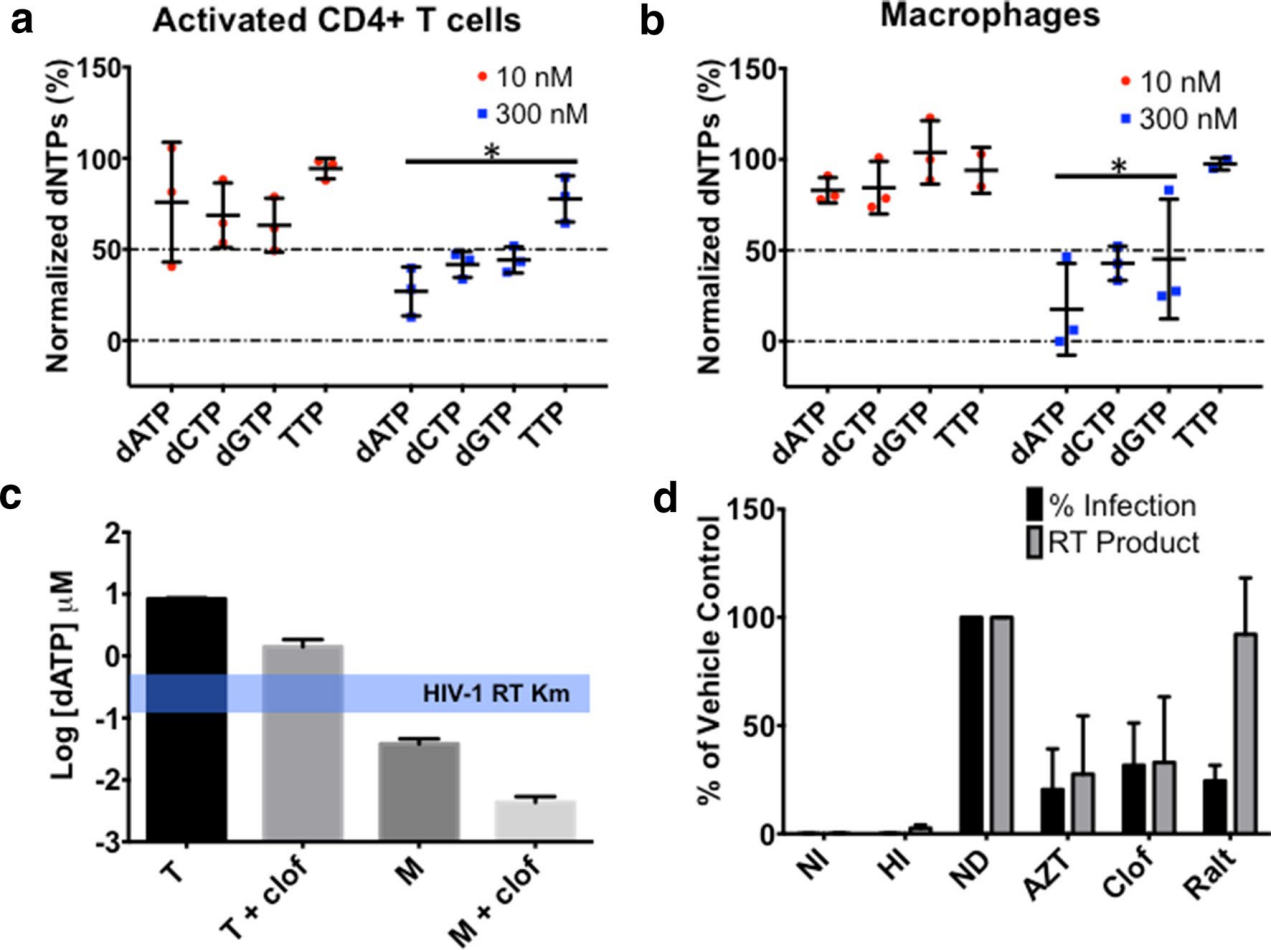
Clofarabine induced depletion of cellular dNTPs and inhibition of reverse transcription. Effect of clofarabine on cellular dNTP levels in primary activated CD4^+^ T cells (**a**) and macrophages (**b**) from three healthy donors. Activated CD4^+^ T cells and macrophages were treated with the indicated concentration of clofarabine for 8 h, washed with PBS, and dNTPs were extracted for analysis. dNTP levels are expressed as a percentage of the vehicle control (DMSO). Each value represents an individual donor with mean ± SD indicated. *p < .05 compared to vehicle control (multiple t test with Holm-Sidak post hoc test). **c** Cellular dATP concentrations of untreated and clofarabine (300 nM) treated T cells and macrophages from three healthy donors. Measured dATP levels were converted to cellular dATP concentrations as previously described [8], and are expressed as mean ± SD).* Blue bar* indicates the Km range of HIV-1 RT [8, 31]. T: untreated T cells, T + clof: T cells + 300 nM clofarabine, M: untreated macrophages, M + clof: macrophages + 300 nM clofarabine. **d** Clofarabine effect on HIV-1 proviral DNA synthesis. MAGI cells were incubated with DMSO (NI, HI, ND), 200 nM AZT, 300 nM clofarabine (Clof), or 50 nM raltegravir (Ralt) for 2 h prior to infection with pseudotyped HIV-1. Infection (*black bars*) was determined at 48 h post-infection, and reverse transcription (*grey bars*) efficiency was determined at 18 h post-infection. Data represent mean ± SD from three independent experiments and are expressed as percentage of vehicle control (DMSO, 100 %). *NI* no infection, *HI* heat inactivated virus, *ND* no drug

We pretreated both cell types, from three healthy donors with two different clofarabine concentrations, 10 nM (below IC_50_ values for both cell types, Fig. 1b and c) or 300 nM (above IC_50_, but below CC_50_ values for both cell types), and methanol extracted the cellular dNTPs 8 h later. To measure the cellular dNTP levels we utilized a single nucleotide primer extension assay that we previously developed [8]. Briefly, this assay uses a 5′ P^32^ radiolabelled 23-mer primer (P) annealed to one of four distinct 24-mer templates (T). The single nucleotide overhang on the 24-mer template (A, C, G or T) determines the dNTP to be measured. The template/primer was incubated with extracted cellular dNTPs and purified HIV-1 RT. The increase in radiolabelled 24-mer product indicates that the dNTP specific for the template has been incorporated. In three donors, clofarabine induced at least 50 % reduction of dATP, dCTP and dGTP at 300 nM in activated CD4^+^ T cells (Fig. 2a) and macrophages (Fig. 2b). As with many RNRIs, dATP levels were the most affected by clofarabine-induced inhibition of RNR. This is possibly due to inefficient dATP synthesis via the salvage pathway, which does not involve RNR and is not affected by RNRIs [32–34]. Consistent with other reports, TTP levels were the least affected in both cell types possibly due to synthesis of TTP from dCMP and dUMP via the salvage pathway [21, 35]. One caveat of this assay is that clofarabine-TP is present in the cells and could be incorporated by HIV-1 RT. The predicted effect of clofarabine-TP incorporation would be an overestimation in the amount of dATP calculated. Despite this potential problem, we saw a strong depletion of dATP in clofarabine-treated cells. This indicates that although the dATP levels may be overestimated we still observe effective RNR inhibition. We also measured the effect of clofarabine on dNTP levels in MAGI cells (a transformed cell line) using a mass spectrometry-based assay, which would be unaffected by clofarabine triphosphate, and saw a similar dNTP depletion profile to that seen in activated CD4^+^ T cells (Additional file 2: Figure S2A).

As shown in Fig. 2c, the dNTP concentrations in untreated macrophages (M) are already well below the Km of HIV-1 RT, and the clofarabine-induced dNTP reduction (M + clof) would be expected to further delay HIV-1 reverse transcription and inhibit viral infection (as observed in Fig. 1c). However, the dATP concentration in activated CD4^+^ T cells (T) and in clofarabine-treated T cells (T + clof) remains above the Km value of HIV-1 RT, suggesting that the clofarabine-induced dNTP reduction in T cells should not significantly affect HIV-1 reverse transcription kinetics. Despite this, we do see a reduction in pseudovirus infection in CD4^+^ T cells at 300 nM clofarabine treatment (Fig. 1b). One possible explanation is that the toxicity associated with clofarabine treatment may be responsible for the decrease in infection; however, we see potent anti-HIV-1 activity at levels that are not toxic, making this unlikely. Another possibility is it that clofarabine may be acting through an additional mechanism that is not directly related to its RNR inhibition.

Next, we confirmed that the observed clofarabine-induced HIV-1 inhibition (blue lines in Fig. 1b, c) is due to the inhibition of HIV-1 DNA synthesis by employing quantitative PCR to measure a reverse transcription product. Clofarabine treatment led to a decrease in viral DNA synthesis that correlated with a decrease in infection (Fig. 2d). These results are similar to those seen with AZT, a known chain terminator. In contrast, the integrase inhibitor raltegravir (Ralt) decreased infectivity by approximately 80 %, but this loss did not correlate to a reduction in viral DNA synthesis. These results indicate that clofarabine reduces reverse transcriptase-mediated viral DNA synthesis through, at least in part, its inhibition of RNR, which deplete endogenous dNTPs.

### Incorporation of clofarabine-TP by reverse transcriptase

**Fig. 3:**
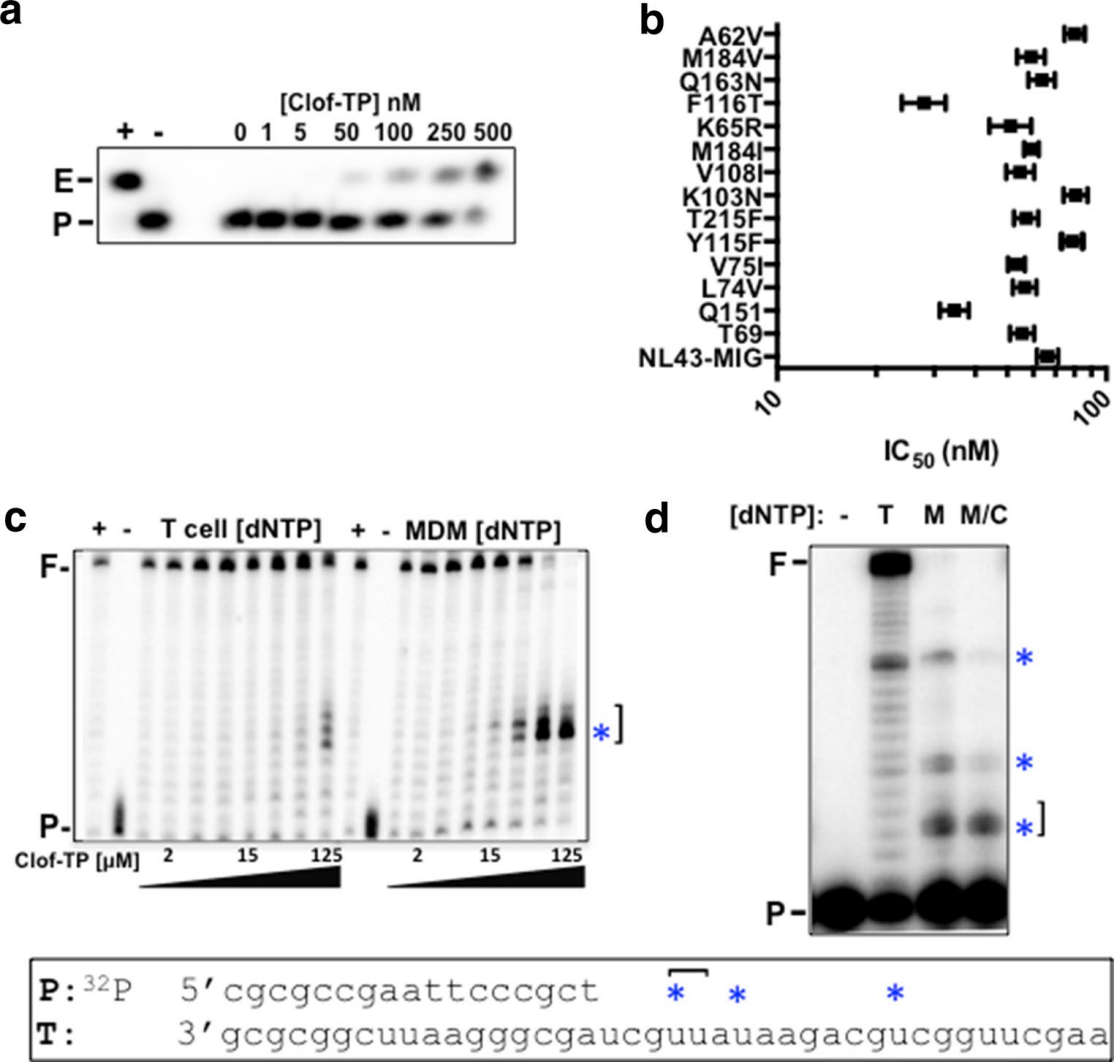
Biochemical examination of the dual mechanism of clofarabine. **a** Direct clofarabine-TP incorporation by HIV-1 RT. A 5′ ^32^P-labeled 23-mer DNA primer (P) annealed to a 24-mer DNA template with a single T overhang was incubated with HIV-1 RT and increasing concentrations of clofarabine-triphosphate (Clof-TP). E, Extended product; +, 50 μM dATP positive control; −, no dATP control. **b** Effect of clofarabine-TP incorporation on DNA synthesis. A 5′ ^32^P-labeled 17-mer DNA primer (P) annealed to a 38-mer RNA template was extended by HIV-1 RT with fixed dNTP concentrations found in either activated T cells (T cell, 5 μM) or monocyte-derived macrophages (MDM, 50 nM) with increasing concentrations of clofarabine-TP (two-fold dilutions starting at 125 μM). F, Fully extended product; +, 50 μM dNTP positive control; −, no dNTP control. *Blue asterisks* (*) indicates the clofarabine-TP incorporation site followed by kinetic pauses (]). **c** Clofarabine inhibition of NRTI-resistant RT mutants. MAGI cells were treated with increasing concentrations of clofarabine for 2 h prior to infection with Vsvg-pseudotyped HIV-1 containing mutations in RT. Flow cytometry analysis for infected cells was conducted at 48–72 h post-infection. IC_50_ values and 95 % confidence intervals are shown. NL4-3 MIG: wild-type HIV-1 RT, Q151: A62V, V75I, F77L, F116Y and Q151M, T69: M41L, A62V, T69S, K70R, T215Y and serine–serine insertion between 69 and 70. **d** Biochemical simulation of HIV-1 RT activity at dNTP concentrations found in cells with and without clofarabine treatment. A 5′ ^32^P-labeled 17-mer DNA primer (P) annealed to a 38-mer RNA template (shown in *box*) was extended using an equal amount of purified HIV-1 RT protein with dNTP concentrations found in T cells (T, 5 μM), macrophages (M, 50 nM) or macrophages treated with 300 nM clofarabine (M/C, 10 nM dATP, 28 nM dCTP, 28 nM dGTP, 40 nM TTP). *Blue stars* (*) indicate kinetic pause sites, F, fully extended 38 bp product; −, no dNTP control

Our activated CD4^+^ T cell data indicated that the clofarabine-induced dNTP depletion would still provide a kinetically favorable environment for reverse transcription. It has been reported that clofarabine triphosphate (clofarabine-TP) is efficiently incorporated into DNA by DNA polymerase α and ε as a dATP analog [21, 22]. Therefore, we tested whether HIV-1 RT can directly incorporate clofarabine-TP into DNA. For this test, clofarabine-TP and purified HIV-1 RT were incubated with a 5′ ^32^P-labeled 23-mer DNA primer (P) annealed to a 24-mer DNA template containing a single T overhang, which allows RT to incorporate a single clofarabine-TP. As shown in Fig. 3a, a 24-mer extended product (E) was observed in the presence of clofarabine-TP at concentrations as low as 50 nM and the amount of 24-mer produced increased with increasing concentrations of clofarabine-TP. These results demonstrate that clofarabine-TP is a substrate for HIV-1 reverse transcriptase. HIV-1 RT is known for its high error rate and low fidelity [36, 37], raising the possibility that clofarabine could act as a general purine nucleoside analog. Therefore, we also tested whether clofarabine could be incorporated as a dGTP analog by using a “C” overhang template/primer. In this assay, we did not observe any clofarabine-TP incorporation by HIV-1 RT (data not shown), indicating that clofarabine-TP is incorporated only as a dATP analog, not as a nonspecific purine analog.

Next, we investigated whether the incorporation of clofarabine-TP by HIV-1 RT inhibits primer extension. For this experiment, reactions contained 5′ ^32^P-labeled 17-mer primer (P) annealed to a 38-mer RNA template (sequence indicated below, Fig. 3 B/D in box), dNTPs (at either the concentrations found in activated T cells, 5 μM, or macrophages, 50 nM), increasing concentrations of clofarabine-TP, and excess HIV-1 RT. As shown in Fig. 3b, when the primer was extended by HIV-1 RT at the T cell dNTP concentrations (5 μM), pausing of DNA synthesis (* in Fig. 3b) was observed only at the highest dose of clofarabine-TP (125 nM). However, at the macrophage dNTP concentrations (50 nM), the RT pausing was observed at much lower clofarabine-TP concentrations, and the full-length extension was completely inhibited at the highest clofarabine-TP concentration (125 nM). As expected, a stall site was observed across from a U site in the template (* in Fig. 3b) indicating that clofarabine-TP was incorporated as an adenosine analog. More interestingly, this stall induced inefficient subsequent dNTP incorporation (] in Fig. 3b), causing RT pausing and eventually blocking full length DNA synthesis. These data indicate that HIV-1 RT can directly incorporate clofarabine-TP and that its incorporation inhibits processive viral DNA synthesis, particularly at the low dNTP environment found in non-dividing macrophages.

There are numerous mutations in RT that render HIV-1 resistant to NRTIs, many of which confer multi-drug resistance. To determine whether these mutations would also confer resistance to clofarabine, we treated MAGI cells with increasing amounts of clofarabine for 2 h prior to infection with pseudotyped HIV-1 vectors containing a panel of known RT mutations [38]. As shown in Fig. 3C, clofarabine had similar potency against the NRTI-resistant RT mutants as against wild type (NL4-3 MIG). This data could indicate that the primary mechanism of HIV-1 inhibition is RNR inhibition rather than incorporation by RT. However, many mutations in RT that confer resistance to individual NRTIs have no effect on different NRTIs, and some can increase susceptibility to certain drugs. Interestingly, clofarabine’s structure is very different from all FDA-approved NRTIs because it has a 3′OH and is not an obligate chain terminator. It is possible that the potency of clofarabine against known NRTI-resistant RTs is due to its differential chemical structure and that clofarabine may work via both mechanisms: RT and RNR inhibition. Selection experiments to develop a clofarabine-resistant strain are planned to further explore this question.

In order to separate the two distinct mechanisms of clofarabine, reduction of dNTPs and direct inhibition of DNA synthesis via incorporation, we biochemically compared the DNA synthesis efficiency of HIV-1 RT at the cellular dNTP concentrations described in Fig. 2c. For this, we extended a 5′ ^32^P-labeled 17-mer primer (P) annealed to a 38-mer RNA template (sequence indicated in box below Fig. 3b/d) with an equal amount of purified HIV-1 RT protein at three different dNTP concentrations: that found in T cells (T, 5 μM), in macrophages (M, 50 nM) and in macrophages that had been treated with 300 nM clofarabine (M/C, Fig. 3d). This experiment was conducted in the absence of clofarabine so that we could observe the effect of clofarabine induced dNTP reduction on DNA synthesis in the absence of any effects of clofarabine incorporation. As shown in Fig. 3d, HIV-1 RT generated the fully extended product (F) at T cell dNTP concentrations, whereas HIV-1 RT displayed limited primer extension with clear kinetic pausing (*) in the macrophage simulated dNTP concentrations. This RT-pausing was further enhanced at the dNTP concentrations observed in clofarabine treated macrophages, which ranged from 10 to 40 nM (Fig. 3d). These data recapitulate previous data indicating that the dNTP pools in macrophages have a restrictive effect on reverse transcription [8, 14] and support our hypothesis that a further reduction in dNTPs will lead to a corresponding decrease in HIV-1 viral DNA synthesis. From these biochemical analyses, we suggest that clofarabine induced dNTP depletion in macrophages reduces reverse transcription (Fig. 3d) while also increasing the likelihood of direct incorporation of clofarabine-TP during reverse transcription, which in turn can cause inhibition of processive DNA synthesis (Fig. 3b).

**Fig. 4:**
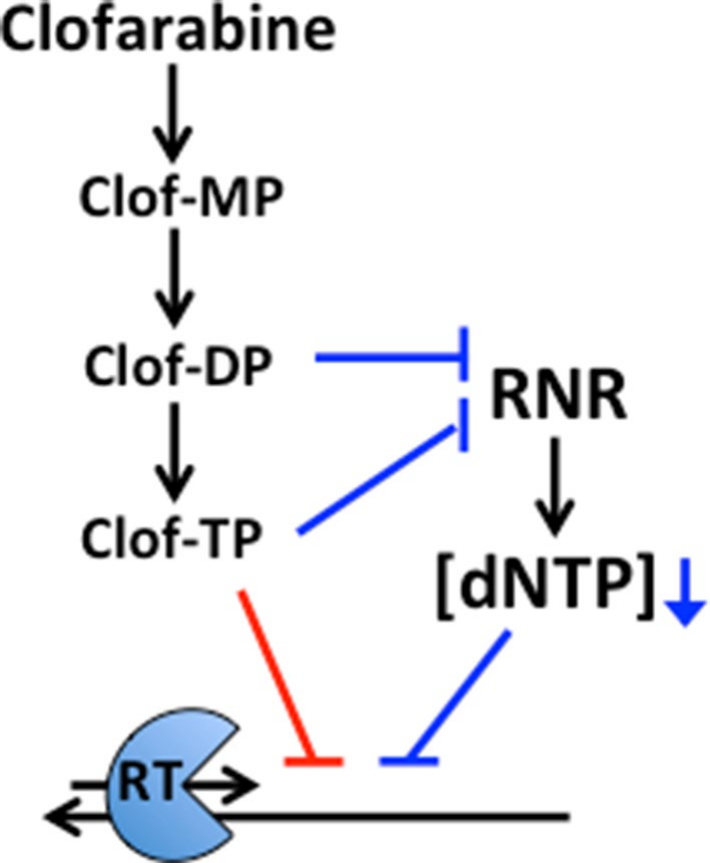
Model for the anti-HIV-1 dual action mechanisms of clofarabine in macrophages. Clofarabine di- and triphosphate inhibit RNR to reduce dNTP levels, leading to the kinetic suppression of HIV-1 reverse transcription (*blue*). Clofarabine triphosphate is also directly incorporated by HIV-1 RT, inhibiting extension (*red*)

As summarized in Fig. 4, we report that clofarabine, which is converted to clofarabine-DP and clofarabine-TP in cells, can inhibit HIV-1 reverse transcription by two distinct mechanisms: (1) reduction of cellular dNTPs through the inhibition of RNR (by clofarabine-DP and clofarabine-TP) and (2) direct incorporation of clofarabine-TP by HIV-1 RT and inhibition of viral DNA synthesis. Importantly, the inhibitory impact of clofarabine imposed by these two mechanisms becomes more effective in non-dividing macrophages because this viral target cell type is less sensitive to the toxic effects of clofarabine. In addition, macrophages maintain limited dNTP pools, in part due to the dNTPase activity of host protein SAMHD1. Interestingly, many nucleoside analogs are not substrates for SAMHD1, which increases nucleoside reverse transcriptase inhibitor (NRTI) efficacy in macrophages [39]; however, it was recently published that clofarabine-TP is degraded by SAMHD1 in vitro [25]. Despite this, clofarabine was still able to reduce dNTPs and restrict viral replication efficiently in macrophages.

Our previous publication reported that clofarabine is threefold more effective against HIV-2 than HIV-1 [23]. This result is surprising in light of our current report that clofarabine exerts its antiviral effect at least in part through reducing dNTP levels: the HIV-2 protein Vpx counteracts SAMHD1 and promotes retroviral replication in macrophages by elevating cellular dNTPs, and therefore would be expected to be more resistant to clofarabine than HIV-1, which does not contain Vpx. However, the previous work on HIV-2 was performed in U373-MAGI-CXCR4_CEM_ cells, a transformed cell line derived from a human glioblastoma, which have very limited SAMHD1 expression, abundant dNTPs and are highly permissive to HIV-1 infection. This indicates that the difference seen between HIV-1 and HIV-2 inhibition by clofarabine in the previous report is likely not Vpx or dNTP related but more likely related to differences in RT fidelity. Comparing the effect of clofarabine on HIV-1 and HIV-2 in primary human macrophages would be interesting, but we believe it would be difficult to explain the outcome mechanistically due to the SAMHD1-mediated hydrolysis of clofarabine-TP (31). Degradation of SAMHD1 by Vpx would lead to an increase in both dNTP and clofarabine-TP levels complicating the expected experimental outcome of the potency of clofarabine. A dual RNRI/NRTI that is resistant to SAMHD1 hydrolysis would be expected to have significantly higher efficacy against HIV-1 than does clofarabine, and would be a much better tool to study the effects of Vpx on RNR inhibition.

Any suggestion of using an RNRI as a clinical treatment for HIV-1 runs into the specter of hydroxyurea, an RNRI which failed clinically as an anti-HIV drug due primarily to its accentuation of NRTI toxicity [40]. Given the self-potentiation inherent to clofarabine’s dual mechanisms of action, potentiation of toxicity would be a serious risk; however, there are significant differences between the two RNRIs. Hydroxyurea and clofarabine differ greatly in structure and in the mechanism of RNR inhibition. Clofarabine has been used as a second line cancer treatment since its FDA approval in 2004 and recent trials have had positive results using it as a first line cancer treatment that can be administered orally [41, 42]. Any use of clofarabine as an HIV drug would require further drug interaction studies to establish the safety of clofarabine both alone and with commonly used combination therapies. The current study was designed to study the mechanism of action and toxicity of clofarabine as a model for dual RNR/HIV-1 RT inhibitors in primary human target cells. We propose that clofarabine be used as a base molecule for further drug design studies. A derivative that shows resistance to SAMHD1 hydrolysis while maintaining the activity and low toxicity of clofarabine in macrophages would be of great interest. This would increase drug efficacy by reducing the natural dNTP competition and ensure that drug concentrations remain high for increased incorporation. In conclusion, our experimental observations of cellular dNTP level reduction by clofarabine (below Km value of HIV-1 RT) and more effective incorporation of clofarabine-TP at these lowered dNTP concentrations support that clofarabine can be a model agent for dual function anti-HIV agents.

## Methods

### Cell lines, plasmids, and chemicals

The 293T cell line was obtained from the American Type Culture Collection (Manassas, VA) while U373-MAGI-CXCR4_CEM_ (MAGI) cells were obtained from Dr. Michael Emerman through the AIDS Research and Reference Reagent Program, Division of AIDS, NIAID, NIH [43, 44]. The HIV plasmid pNL4-3 MIG has been previously described [24]. RT resistant mutants were created as previously described [38] and subcloned into pNL4-3 MIG. The VSV-G envelope plasmid, pHCMV-VSVG, was provided by J. Burns (University of California, San Diego). Clofarabine was purchased from Carbosynth (Berkshire, UK). The following reagents were obtained from the NIH AIDS Reagent Program, Division of AIDS, NIAID, NIH: Raltegravir (Cat # 11680) from Merck & Company, Inc. and zidovudine.

### Cell culture

293T and MAGI cells were maintained as previously described [45]. Primary human monocytes and CD4^+^ T cells were isolated from peripheral blood buffy coats by positive selection using CD14 or CD4 beads (Miltenyi Biotec, San Diego, CA), as previously described [46], and maintained in RPMI medium with 10 % FCS and penicillin/streptomycin. CD14^+^ monocytes were matured to macrophages using 5 ng/mL human GM-CSF for 7 days (Miltenyi Biotec). CD4^+^ T cells were activated with 5 µg/mL phytohemagglutinin (Sigma-Aldrich) and 20 units/mL IL2 (Miltenyi Biotec) for 1 day, then 20 units/mL IL2 alone for 5 days.

### Production of viral stocks

293T cells were co-transfected with pNL4-3 MIG and pHCMV-VSVG using linear polyethylenimine from Polysciences, Inc. (Warrington, PA) as previously described [47]. Virus was harvested at 48 and 72 h post-transfection and used immediately or frozen at −80 °C.

### Drug treatments and infections

MAGI cells were infected as described previously [45]. Cells were treated with clofarabine for 2 h prior to infection with viral supernatant. Cells were harvested for analysis 48–72 h after infection and analyzed with a BD Biosciences LSRII flow cytometer. Vehicle treated cells had 15–35 % infection. Primary cells were treated with clofarabine for 8 h, washed twice with PBS and then infected with viral supernatant. To achieve the same infectivity, MDMs were infected with five times the amount of viral supernatant than T cells. Monocyte derived macrophages (MDM) were collected 5 days post-infection and activated CD4^+^ T cells were collected 3 days post-infection for analysis via flow cytometry. Data analysis was performed using FloJo and Prism 6. IC_50_ and CC_50_ were determined using GraphPad Prism nonlinear fit analyses; log (inhibitor) versus response–variable slope.

### Cell proliferation and cytotoxicity

MDMs and T cells were treated with clofarabine for 8 h, washed twice with PBS and maintained in media 5 or 3 days, respectively (same treatment as infection protocol). The XTT assay from ATCC was used as per the manufacturer’s protocol. The optimized assay incubation time for both cell types was 5 h. For additional measures of toxicity, including trypan blue exclusion and cell size, cells were treated the same as above and analyzed using the Vi-Cell Counter (Beckman Coulter).

### Real-time qPCR of RT products

Real time qPCR was performed essentially as previously described [48]. MAGI cells (150,000 per well) were plated on a six-well plate. Twenty-four hours later, cells were treated with the indicated drug for 2 h prior to infection. As a control, an aliquot of virus was heat inactivated for 30 min at 95 °C. Eighteen hours after infection, cells were harvested with trypsin; half were re-plated for the analysis of infection, and DNA was isolated from remaining cells using HighPure PCR Template Preparation Kit (Roche, Basel, Switzerland). Quantitative PCR (qPCR) mixtures contained 4 μL of eluted DNA using iTaq Universal SYBR Green Supermix according to the manufacturer’s suggestions (BioRad, Hercules, CA). Primers for 18S rRNA were used to normalize sample-to-sample variation. The primers used to detect late reverse transcription (RT) products were 5′TGTGTGCCCGTCTGTTGTGT (forward) and 5′GAGTCCTGCGTCGAGAGAGC (reverse). The primers used to detects 18S rRNA were 5′GTAACCCGTTGAACCCCATT (forward) and 5′CCATCCAATCGGTAGTAGGG (reverse). The conditions for amplification were 95 °C for 10 min, followed by 40 cycles of 95 °C for 30 s, 60 °C for 30 s, and 72 °C for 30 s.

### Synthesis of clofarabine triphosphate

Clofarabine triphosphate was synthesized as previously described [49] with minor modifications. In separate round-bottom flasks, clofarabine (0.1 g, 1.0 eq.) and tributylammonium pyrophosphate (0.361 g, 2.0 eq.) were dried under high vacuum for 1 h at ambient temperature. Throughout the entire experiment, the reaction was maintained under an argon atmosphere. Clofarabine (0.1 g, 1.0 eq.) was dissolved in anhydrous dioxane (0.9 mL, 3 mL/mmol) and anhydrous pyridine (0.3 mL, 1 mL/mmol). The flask was cooled with an ice bath. A solution of 2-chloro-4H-1,3,2- benzodioxaphosphorin-4-one (80 mg, 1.2 eq.) in anhydrous dioxane (0.4 mL, 1 mL/mmol) was added and the mixture was then stirred at room temperature for 15 min. A solution of tributylammonium pyrophosphate (361 mg, 2.0 eq.) in anhydrous DMF (0.7 mL, 1 mL/mmol) was added to the mixture, prior to quick addition of tributylamine (0.4 mL, 5.0 equiv). The mixture was stirred for 15 min at room temperature. A solution of iodine (1 % solution in pyridine/water, 9:1) was then added dropwise until the permanent brown color of iodine persisted, and the mixture was stirred for 20 min. The excess of iodine was quenched with a 5 % aqueous solution of Na_2_S_2_O_3_. The reaction mixture was evaporated to dryness under vacuum, dissolved in 25 % ammonia solution, and stirred for 1 h at room temperature. The reaction was monitored by TLC (R_f_ 0.1 isopropanol/aqueous ammonia = 1/1 and MS). The reaction mixture was concentrated under reduced pressure and resulting crude product was purified on a short pad of silica gel with a gradient of isopropanol/aqueous ammonia 8/2 to 1/1 affording the desired 5′-triphosphate ammonium salt, 21 % yield (75 % pure with diphosphate as an impurity, determined by ^31^P NMR). The synthesized nucleoside 5′-triphosphate was confirmed by ^1^H NMR, ^31^P NMR, and HR-MS analyses consistent with published analytical data for clofarabine 5′-triphosphate [19].

### HIV-1 RT purification

HIV-1 (NL4-3) RT homodimer with a hexahistidine tag was expressed using an overexpression system in BL21 *E. coli* [50, 51], and purified using Ni^2+^ chelation chromatography as described previously [8, 51].

### dNTP quantification

dNTPs from primary cells were extracted and quantified using the protocol previously described by Diamond et al. [8]. Briefly, this assay uses a 5′ P^32^ radiolabelled 23-mer primer (P) annealed to one of four distinct 24-mer templates (T). The single nucleotide overhang on the 24-mer template (A, C, G or T) determines the dNTP to be measured. The template/primer was incubated with extracted cellular dNTPs and purified HIV-1 RT for 5 min at 37° and then quenched with 40 mM EDTA and 99 % (vol/vol) formamide at 95 °C for 2 min. The reactions were resolved on a 20 % urea-PAGE gel (American Bio Sequel NE reagent) and imaged using Pharos FX molecular imager (Biorad). The increase in radiolabelled 24-mer product indicates that the dNTP specific for the template has been incorporated. MAGI cells were treated with 100 or 300 nM clofarabine for 8 h, then harvested, counted, resuspended in 60 % methanol, and stored at −20 °C for 18 h. Samples were then vortexed, heated to 95 °C for 3 min, and centrifuged at 16,000×*g* for 5 min. Supernatant was transferred to new tubes and dried in an Eppendorf Vacufuge. Samples were then stored at −80 °C.

To perform LC–MS/MS analysis, dried extracts were reconstituted in water at a concentration of 1 million cells per 100 µL. For each reconstituted sample, a 50 µL aliquot of resuspension was added to an Eppendorf tube containing 50 µL of internal standard (10 µM 5-iodo-dCTP in water), and then diluted with 100 µL water. The samples were then centrifuged at 14,000 rpm for 5 min at 4 °C. LC–MS/MS was used to determine levels of TTP, dGTP, dCTP and dATP in the supernatants following a previous published method [52] with minor modifications. The LC–MS/MS system consists of an AB Sciex QTrap 5500 mass spectrometer and an Agilent 1260 Infinity HPLC. The chromatographic separation of analytes was achieved using a Thermo Scientific Hypercarb column (100 × 3 mm, 5 µm). The two eluents were: (A) 0.5 % diethylamine in water, pH adjusted to 10 with acetic acid; and (B) 50 % acetonitrile in water. The mobile phase was delivered at a flow rate of 0.5 mL/min using stepwise gradients of A and B: 0–20 min, 0–25 % B (v/v); 20–28 min, 25–50 % B (v/v); 28–28.5 min, 50–95 % B (v/v); 28.5–30.5 min, 95–95 % B (v/v); 30.5–31 min, 95–0 % B, (v/v); 31–39 min, 0–0 % B (v/v). Only eluate from 10-30 min was diverted into the mass spectrometer for analysis. MS/MS detection of the analytes was conducted using an ESI ion source with MRM detection in negative mode. The curtain gas was set at 20 psi. The ionspray voltage was set at −4500 V, and the temperature at 650 °C. The nebulizer gas (GS1) and turbo gas (GS2) were both set at 45 psi.

### In vitro clofarabine triphosphate incorporation assay

This single nucleotide extension assay was modified from a previously described assay [51]. A 5′ ^32^P-labeled DNA 18-mer (5′-GTCCCTCTTCGGGCGCCA-3′) was annealed to a 19-mer DNA template (3′-CAGGGAGAAGCCCGCGGTG-5′) at a 1:2 ratio. Extension from an 18-mer to 19-mer indicates that clofarabine triphosphate has been incorporated by HIV-1 reverse transcriptase. 20 μL reactions contained 200 fmol template/primer, 2 μL clofarabine triphosphate at concentrations indicated or 50 μM of dNTPs for the positive control, 4 μL of purified RT (HIV-1 NL4-3), 25 mM Tris–HCl, pH 8.0, 2 mM dithiothreitol, 100 mM KCl, 5 mM MgCl2, and 10 µM oligo(dT). Reactions were incubated at 37 °C for 5 min and then quenched with 10 µL of 40 mM EDTA and 99 % (vol/vol) formamide at 95 °C for 2 min. The reactions were resolved on a 20 % urea-PAGE gel (American Bio Sequel NE reagent) and imaged using Pharos FX molecular imager (Biorad).

### Primer extension assay

An HIV-1 RT primer extension assay was performed as previously described with slight modifications [53]. A 5′ ^32^P-labeled 17-mer DNA primer (5′-CGCGCCGAATTCCCGCT-3′) was annealed to a 40-mer RNA template (5′-AAGCUUGGCUGCAGAAUAUUGCUAGCGGGAAUUCGGCGCG-3′) in the presence of 100 mM NaCl, 10 mM Tris–HCl (pH 8.0) and 1 mM EDTA. 20 µL Reaction mixtures contained 10 nM template-primer, 4 µL of purified HIV-1 RT, 5 µM or 50 nM of all four dNTPs (ThermoScientific), 12.5 mM Tris–HCl (pH 7.5), 12.5 mM NaCl, 2.5 mM MgCl_2_ and 20 µM oligo(dT). Reactions were initiated upon addition of RT and incubated at 37 °C for 1 h (for Fig. 3b) and 5 min (for Fig. 3d). Reactions were terminated with 10 µL of 40 mM EDTA, 99 % formamide and the products were resolved on a 14 % urea-PAGE gel (American Bio Sequel NE reagent) and imaged using Pharos FX molecular imager (Biorad) and analyzed using ImageLab software.

## Additional files

10.1186/s12977-016-0254-0 Infectivity and toxicity in T cells and macrophages. **(A)** Activated CD4^+^ T cells and macrophages were infected with pseudotyped HIV-1. In order to achieve similar levels of infection more pseudovirus was used to infect macrophages (5X more virus used). Individual donor infectivity is shown with technical triplicates shown as standard error (mean with standard deviation). **(B)** Cell viability in activated CD4^+^ T cells. Cells were treated with varying amounts of clofarabine for 8 h, washed in PBS and maintained in media for 72 h. Cell viability was determined by exclusion of trypan blue indicating membrane integrity by the Vi-cell counter. **(C)** Cell size of activated CD4^+^ T cells treated with clofarabine. Cells were treated with varying amounts of clofarabine for 8 h, washed in PBS and maintained in media for 72 h. Cells were counted and cell size was determined using the Vi-Cell counter. **(D)** Cell viability in macrophages. Cells were treated with varying amounts of clofarabine for 8 h, washed in PBS and maintained in media for 5 days. Cell viability was determined by exclusion of trypan blue indicating membrane integrity by the Vi-cell counter. **(E)** Cell size of activated CD4^+^ T cells treated with clofarabine. Cells were treated with varying amounts of clofarabine for 8 h, washed in PBS and maintained in media for 5 days. Cells were counted and cell size was determined using the Vi-Cell counter.
10.1186/s12977-016-0254-0 Clofarabine induced depletion of cellular dNTPs in MAGI cells. MAGI cells were treated with 100 nM (~IC50) and 300 nM (~IC90) clofarabine for eight hours, washed with PBS, and dNTPs were methanol extracted and analyzed by LC–MS/MS. Data shown represents mean ± SD of three independent experiments, and is expressed as a percentage of vehicle control. * = p < .05 compared to vehicle control (multiple t test with Holm-Sidak post hoc test).

## Abbreviations

RT: reverse transcriptase
dNTP: deoxynucleotide triphosphate
RNR: ribonucleotide reductase
IC_50_: half maximal inhibitory concentration
CC_50_: half cytotoxic concentration
SAMHD1: sterile α motif and histidine aspartic domain containing protein 1
dNTP: deoxyribonucleotide triphosphate
RNRI: ribonucleotide reductase inhibitor
NRTI: nucleoside reverse transcriptase inhibitor
MAGI: U373-MAGI-CSCR4_CEM_
MDM: monocyte derived macrophage
EGFP: enhanced green fluorescent protein
AZT: zidovudine
