# Supplementary figures and images for: Dual anti-HIV mechanism of clofarabine
Source: Retrovirology. 2016 Mar 24;13:20. doi: 10.1186/s12977-016-0254-0 (PMC4806454; doi:10.1186/s12977-016-0254-0)

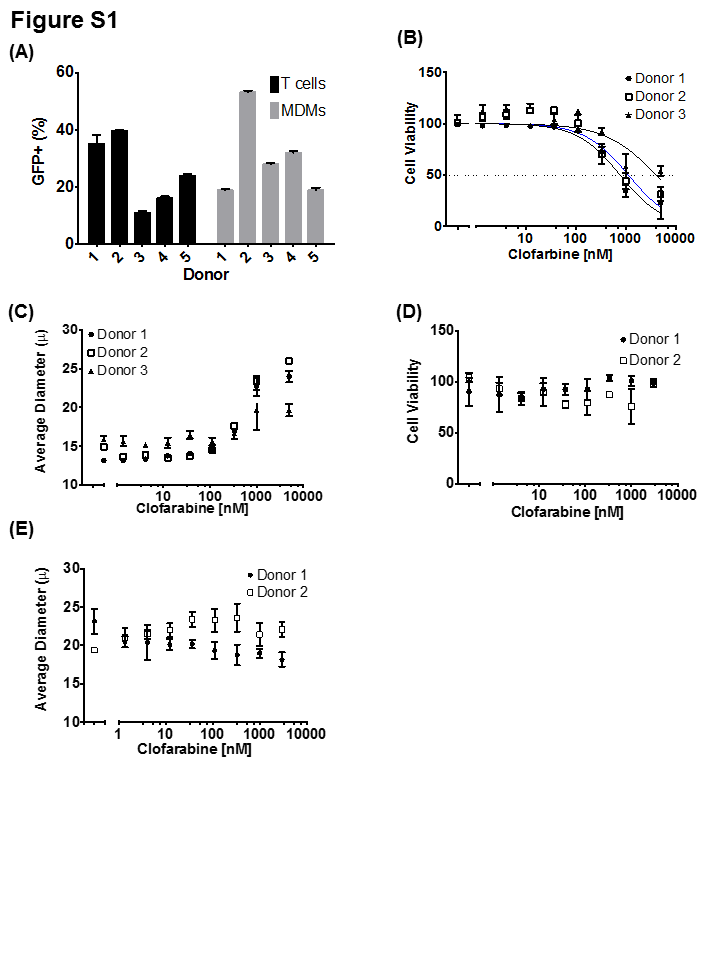

Supplement: Supplementary file 1 — 10.1186/s12977-016-0254-0 Infectivity and toxicity in T cells and macrophages. (A) Activated CD4+ T cells and macrophages were infected with pseudotyped HIV-1. In order to achieve similar levels of infection more pseudovirus was used to infect macrophages (5X more virus used). Individual donor infectivity is shown with technical triplicates shown as standard error (mean with standard deviation). (B) Cell viability in activated CD4+ T cells. Cells were treated with varying amounts of clofarabine for 8 h, washed in PBS and maintained in media for 72 h. Cell viability was determined by exclusion of trypan blue indicating membrane integrity by the Vi-cell counter. (C) Cell size of activated CD4+ T cells treated with clofarabine. Cells were treated with varying amounts of clofarabine for 8 h, washed in PBS and maintained in media for 72 h. Cells were counted and cell size was determined using the Vi-Cell counter. (D) Cell viability in macrophages. Cells were treated with varying amounts of clofarabine for 8 h, washed in PBS and maintained in media for 5 days. Cell viability was determined by exclusion of trypan blue indicating membrane integrity by the Vi-cell counter. (E) Cell size of activated CD4+ T cells treated with clofarabine. Cells were treated with varying amounts of clofarabine for 8 h, washed in PBS and maintained in media for 5 days. Cells were counted and cell size was determined using the Vi-Cell counter. [file 12977_2016_254_MOESM1_ESM.tiff]

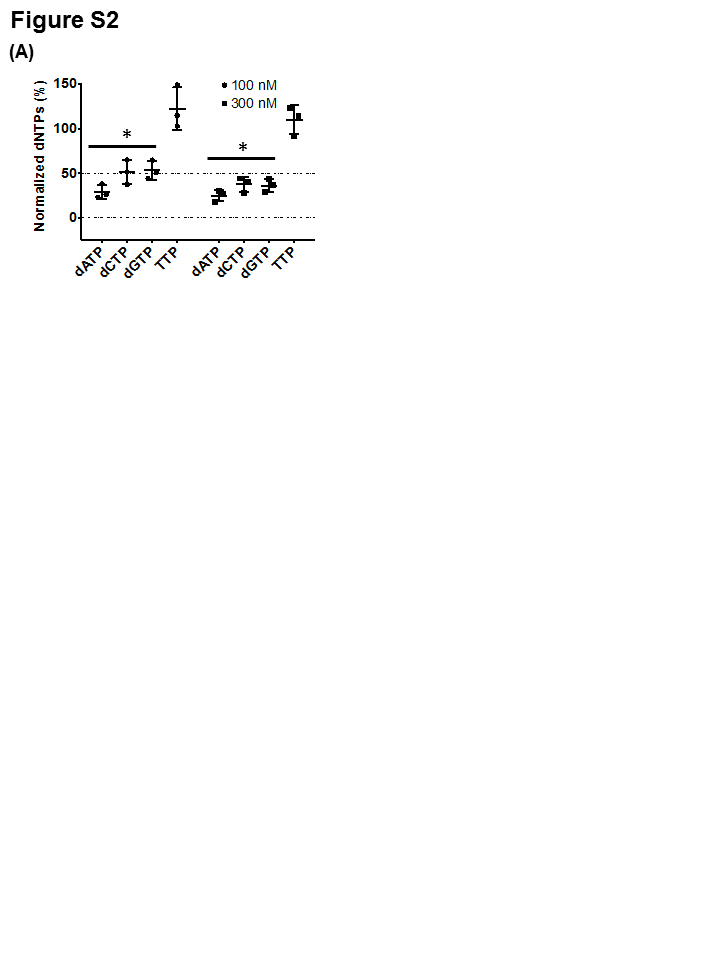

Supplement: Supplementary file 2 — 10.1186/s12977-016-0254-0 Clofarabine induced depletion of cellular dNTPs in MAGI cells. MAGI cells were treated with 100 nM (~IC50) and 300 nM (~IC90) clofarabine for eight hours, washed with PBS, and dNTPs were methanol extracted and analyzed by LC–MS/MS. Data shown represents mean ± SD of three independent experiments, and is expressed as a percentage of vehicle control. * = p < .05 compared to vehicle control (multiple t test with Holm-Sidak post hoc test). [file 12977_2016_254_MOESM2_ESM.tiff]
